# Effect of monopolar diathermy power settings on postoperative pain, wound healing, and tissue damage after tonsillectomy: a randomized clinical trial

**DOI:** 10.1038/s41598-023-50633-z

**Published:** 2024-01-02

**Authors:** Ju Hyun Yun, Jeon Yeob Jang, Yoo Seob Shin, Hyun Jun Kim, Chul-Ho Kim, Do-Yang Park

**Affiliations:** 1 Department of Otolaryngology, Ajou University School of Medicine, 164 Worldcup-ro, Yeongtong-gu, Suwon, 16499 Republic of Korea; 2 Sleep Center, Ajou University Hospital, Suwon, Republic of Korea

**Keywords:** Clinical trial design, Translational research, Adverse effects, Pain management, Surgery

## Abstract

This study aimed to assess the impact of varying monopolar diathermy power settings on postoperative pain, hemorrhage, and wound healing following tonsillectomy. A single-center, prospective, randomized, double-blinded, controlled clinical study was conducted. During bilateral tonsillectomy procedures, one tonsil received low-power settings (15 W, cutting/blend) while the other tonsil received high-power settings (35 W, cutting/blend). Postoperative pain scores (0–10) and wound healing scores (0–3) were evaluated immediately after surgery and at 1, 2, and 4 weeks postoperatively using the visual analog scale. Additionally, histological analysis was performed on electrically resected tonsil tissues to assess tissue damage in the tonsil bed. The allocation of high and low power settings to each side was randomized. Results showed that 1 week after the surgery, the high-power group experienced significantly higher pain scores (mean ± standard deviation: 4.84 ± 2.21) compared to the low-power group (3.56 ± 2.24, p = 0.049). Moreover, the high-power side exhibited slower wound healing during the initial 1–2 weeks postoperatively, as indicated by lower wound scores at 2 weeks (high-power: 1.96 ± 0.64; low-power: 2.43 ± 0.59, p = 0.008). Furthermore, histological analysis revealed significantly deeper tissue degradation on the high-power side compared to the low-power side (p < 0.001), with mean depths of 565.2 ± 291.0 µm and 156.0 ± 36.8 µm, respectively. In conclusion, these findings suggest that when employing monopolar diathermy in tonsillectomy, lower power settings can lead to improved outcomes in terms of postoperative pain, wound healing, and tissue damage.

**Trial registration:** CRIS identifier: KCT0005670 (cris.nih.go.kr, registration date: 11/12/2020).

## Introduction

Tonsillectomy is a multi-frequency surgery with indications for various causes and diseases, such as frequent tonsillitis, obstructive sleep apnea, deep neck infection, and oropharyngeal abscess^1–3^. Since the conception of tonsillectomy, resection and hemostasis of the tonsils have utilized a variety of methods, from tools such as thread, surgical steel knives, and traditional compression dressing to various suture techniques. Early tonsillectomy was time-consuming and associated with a relatively large amount of perioperative bleeding and delayed wound recovery^4^. From the mid-1900s to the present day, electrical surgical units (ESUs) capable of relatively rapid surgery and hemostasis, such as electric monopolar cautery^4,5^, coblation^6^, and plasmablade^7^, have been applied in tonsillectomy. These innovations aim to reduce operation time, minimize complications, decrease intraoperative and postoperative bleeding, alleviate postoperative pain, and shorten the recovery period^8^. The most commonly used ESU device is a monopolar electro-surgery instrument^9–11^.

There have been various studies and guidelines for tonsillectomy, mainly in terms of postoperative major and minor bleeding and reoperation. Representatively, in 2004, Lowe et al. observed that when monopolar diathermy was applied, it showed a statistically significant increase in postoperative bleeding and reoperation rates compared to cold steel knife and the NICE guideline published in 2005 reported that it was better to avoid tonsil surgery using monopolar diathermy^12,13^. However, there have been differences of opinion on the study design^14^, and there have also been various opposing studies on the results^15,16^. Even after that, monopolar diathermy for tonsillectomy is the most frequently applied method^4,11,16–18^. It may be caused by differences in the medical environment between countries, shorter surgery time and convenience of surgery, which can be related to the rate of complications in patients. In addition, since most studies focused on bleeding and reoperation, few studies reported on patient discomfort, pain, or wound recovery.

According to the results of tonsillectomy studies performed with various instruments with various amounts of energy, it is thought that high bleeding and reoperation tendencies are mainly observed when devices with high energy are used^2,19–21^. Monopolar electro-surgery instrument can adjust the electrical power (0–300 W) within the allowable range, and support three or more modes depending on the electrical frequency and type. The mode is determined according to the target area, the depth of electrical penetration, and whether hemostasis should be performed simultaneously. Regarding electrical power (Watt) for cutting, the operator should begin by using the minimum predictable power and gradually increase it to determine the optimal power^22,23^. However, in most cases, the initial power is determined by the experience of the surgeon and the routine manual supplied by the hospital, and the ESU power is finely adjusted at the discretion of the surgeon during surgery.

Despite the need for objective research, few well-controlled systematic studies of the relationship between ESU power and postoperative parameter, such as discomfort, pain, and wound healing parameters, have been conducted. Therefore, even in monopolar electro-surgery, postoperative results such as the recovery rate of the surgical wound and patient pain can differ according to the applied ESU power. However, the effects of varying monopolar diathermy power settings on postoperative parameters including patient outcome and histological characteristics have not been established.

We studied the patient’s pain levels, wound healing rates, and tissue status according to the amount of energy used in monopolar diathermy, which has been the most commonly used method until now. This study aimed to investigate the effect of varying monopolar diathermy power settings on postoperative pain, hemorrhage, complications, and wound healing following tonsillectomy.

## Methods

### Study participants

Among the patients who visited our hospital between Dec 2018 and September 2020, we enrolled participants between 19 and 65 years old who required bilateral tonsillectomy for indications such as chronic frequent tonsillitis, tonsillolith, and simple snoring with tonsillar hypertrophy. Children and pregnant women, patients with abnormal enlargement of one side of the tonsils, excessive asymmetry of the left and right tonsils, abscess on one or both sides of the tonsils, high inflammatory findings, and patients requiring radical surgical treatment for which it is difficult to secure adequate surgical planes due to tonsil cancer or related lesions were excluded.

**Figure 1:**
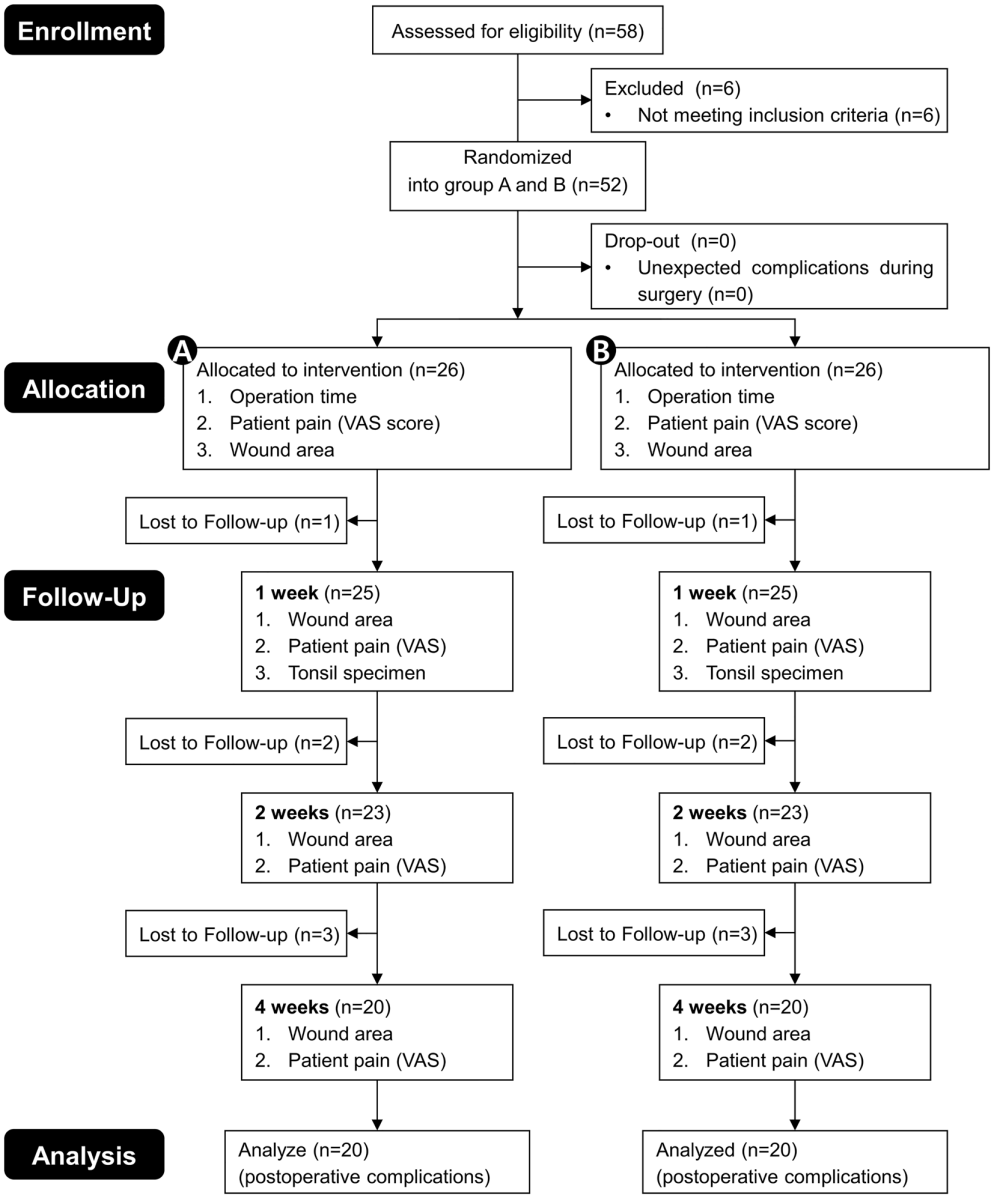
CONSORT flow diagram for study enrollment. *VAS* visual analog scale.

Of the 29 patients who agreed to participate in the study, three were initially excluded due to a history of peritonsillar abscess. In the middle of the study, six patients were excluded due to follow-up loss after discharge. In the final analysis, 40 bilateral tonsils of 20 patients were analyzed, completely (Fig. 1). This prospective, double-blinded, randomized clinical trial was conducted after obtaining approval from the Institutional Review Board of a tertiary hospital (AJIRB-MED-THE-18-398). This clinical trial was registered in the Clinical Research Information Service, Republic of Korea (No. KCT0005670, registration date: 11/12/2020). Written informed consent was obtained from all patients. We conducted this study in accordance with the Helsinki Declaration and the Good Clinical Practice guidelines and reported the findings based on the applicable Consolidated Standard of Reporting Trials guidelines. A reporting guideline checklist is included in the Supplementary Information file.

### Protocol for tonsillectomy with varying electrical power

A single otolaryngologist expert performed the tonsillectomy using a monopolar diathermy unit (Valleylab Force FX Electrical Generator C, Boulder, CO) and blend mode with a sharp steel needle tip (Covidien‐Medtronic, Minneapolis, MN) with different electrical intensities applied to the left and right sides. Among the monopolar electro-surgery intensities, mainly adjusted at 20 W or less^17^, that are generally recommended in the official manual (10–40 W)^23^, we excluded the minimum (10 W) and maximum (40 W) values. These two extreme values were not applied in order to avoid possible complications in the participants. A simple randomization method was used: an online randomization program (http://www.randomization.com) was used to determine the which power (35 W or 15 W) was applied to which side for each patient, i.e. 15 W on one side and 35 W on the other. In the event of unexpected excessive bleeding, damage to the tonsil capsules, unexpected tonsil abscesses, or other complications during surgery, it was decided to apply the conventional electrical power of 20 W and drop out the participants from the study. Hemostasis was performed to a minimum degree when bleeding occurred with 15 W power using a bipolar instrument.

### Outcome measures and follow-up

The operation time was measured for each side. The pain was evaluated using the Wong–Baker Pain Scale^24,25^ (a visual analogue scale [VAS] with a score of 0–10) for each side within 2 h and at 1, 2, and 4 weeks postoperatively. Patients who were not informed to which side received which monopolar settings, were asked about their pain level on each side as follows: “On a scale of 0 to 10, where 0 indicates no pain and 10 represents the most severe pain you have ever experienced, what is your pain score?”. Endoscopic evaluations of the surgical wounds on each side were performed within 2 h and at 1, 2, and 4 weeks postoperatively. Postoperative wound healing was evaluated by another otorhinolaryngologist expert who did not perform tonsillectomy and was blinded to patient information, by scoring the size of pinkish membrane coverage over the tonsillar fossa^26^. Coverage of < 10% = 0 point; 10% ≤ 1 point < 25%; 25 ≤ 2 points < 50%; and 50% ≤  3 points. Postoperative complications were also recorded for each side.

### Histological analysis

The effect of the ESU power on the surgical wound can be accurately determined by measuring the depth of the surgical wound (i.e., of the electrical burn), which can be achieved by harvesting the superior pharyngeal constrictor muscle of the tonsil bed after tonsillectomy. However, in human studies, this is against safety and ethics regulations. Therefore, the degree of surgical burn and depth of electrical penetration were measured by staining the resected side of the tonsils, which is believed to reflect the influence of the electrical burn on the tonsil bed.

In a standardized manner, the tonsil specimens were excised by electro-surgery at 15 W and 35 W. The excised tonsils were fixed in 10% formalin overnight. The tonsils were then embedded in paraffin, and the paraffin blocks were sectioned at 5 μm thickness. To visualize the wounds and connective tissue around the tonsils, the slides were stained with hematoxylin and eosin and Masson’s trichrome. After staining, tonsil specimens were imaged using an inverted light microscope (magnification × 40, IX73-F22PH, Olympus). The dimensions of the cutting plane of the tonsil surface in images were calculated using ImageJ software. The thickness of the connective tissue, which was hypothesized to shrink due to the electrical energy, and the depth of wound damage were measured.

### Statistical analysis

The sample size was calculated using G*Power 3.1.9.2 (Heinrich-Heine-Universität, Germany), with the effect size selected based on both the researcher’s experience and previous literature^26,27^. With paired surgical wound side of high power applied and low power applied, statistical analysis was conducted using paired t-tests. Statistical analysis was conducted using SPSS version 22.0.0 (IBM Corp., Armonk, NY) and GraphPad Prism 6 (GraphPad Software Inc., La Jolla, CA). All data are expressed as the mean ± standard deviation. A *p*-value of < 0.05 (two-sided) was regarded as statistically significant.

### Ethical approval

All procedures performed in the studies involving human participants were in accordance with the ethical standards of the institutional and/or national research committee and with the 1964 Helsinki declaration and its later amendments or comparable ethical standards.

### Informed consent

Informed consent was obtained from all individual participants included in the study.

## Results

### Patient demographics

There were a total of 26 participants, mean participant age was 24.7 ± 5.4 (young adult), the male: female ratio was 9:17, mean BMI was 26.3 ± 4.1 (mild obesity)^28^, and the most common surgical indication was frequent tonsillitis (Table 1).

**Table 1:** Baseline demographic characteristics of enrolled patients (n = 26).

Variables	Total (n = 26)
Age (years), mean ± SD	24.72 ± 5.43
Male:female	9:17
BMI (kg/m^2^)	26.27 ± 4.11
Reason for tonsillectomy, n (%)	
Frequent tonsillitis	21 (80.7)
Tonsilolith	3 (11.5)
Snoring	2 (7.7)

*SD* standard deviation, *BMI* body mass index.

### Intraoperative and early postoperative outcomes

**Figure 2:**
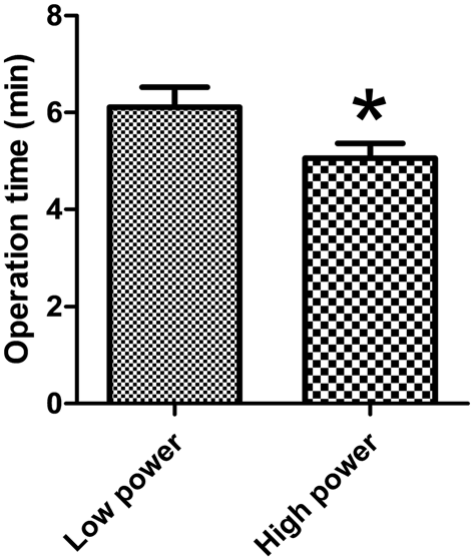
Operation time between high- and low-power settings. The operation time was lower on the high-power side.

**Figure 3:**
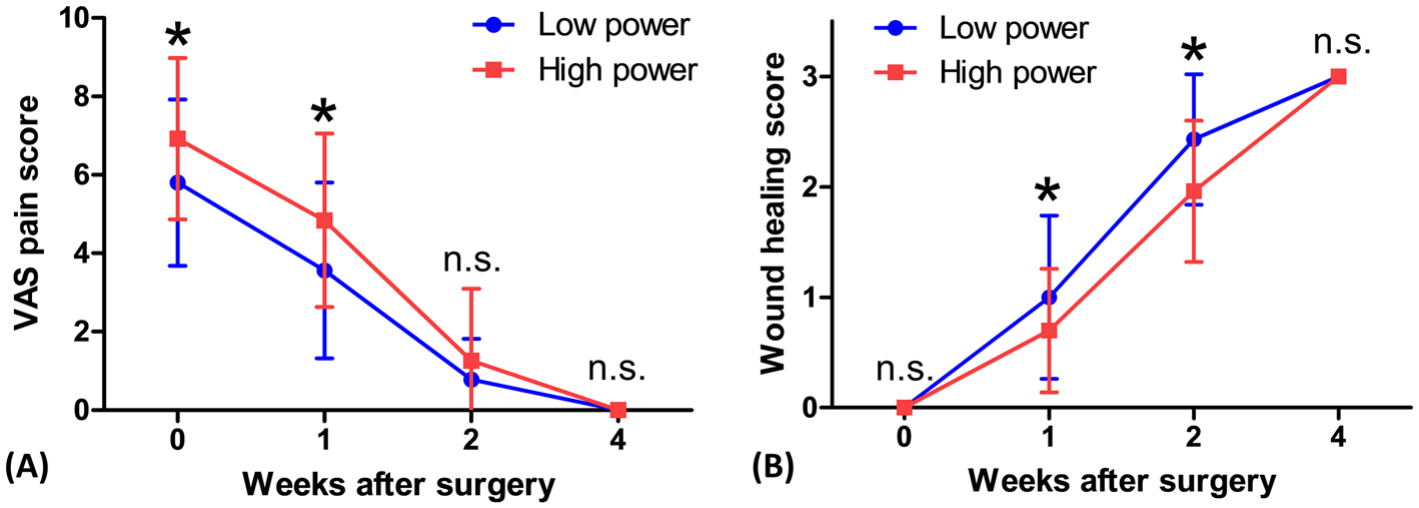
Comparison of visual analog scale pain scores and wound healing scores between the low-power and high-power sides. (**A**) Higher pain was reported on the high-power side than on the lower power side from immediately after surgery to 1 week after surgery. (**B**) The size of the surgical wound was the same immediately after the operation, but at 1–2 weeks postoperatively, wound recovery was slower on the high-power side than on the low-power side. Regardless, both sides were fully healed at 4 weeks. Error bars represent standard deviation. *VAS* visual analog scale, *n.s*. not significant. *p < 0.05.

The operation time was significantly reduced on the high-power side relative to the low-power side, with 5.06 ± 1.54 min in the high-power side group and 6.11 ± 2.06 min in the low-power side group (*p* < 0.001) (Fig. 2). In the evaluation of pain immediately after surgery, the participants’ VAS pain scores were significantly more severe on the side to which high-power was applied, with a 6.92 ± 2.06 pain score in the high-power group and a 5.80 ± 2.12 pain score in the low-power group (*p* = 0.001) (Fig. 3). Since there was no wound healing process right after the surgery, there was no difference in the degree of wound healing in either the high-power or low-power application (Fig. 3). All patients were discharged the day after surgery, and no immediate postoperative bleeding was observed at the surgical site during the admission period.

### Late postoperative outcomes

At week one postoperatively, the pain was significantly greater on the high-power side than on the low-power side, with a 4.84 ± 2.21 pain score in the high-power group and 3.56 ± 2.24 pain score in the low-power group (p = 0.049). At week 2, postoperatively, the pain was not significantly different between the high-power side (1.26 ± 1.84 pain score) and the low-power side (0.78 ± 1.04 pain score, *p* = 0.061). By week 4 postoperatively, there were no pain complaints on either side (Fig. 3). In the comparison of the surgical wound, wound healing was significantly more delayed on the high-power side than on the low-power side with a wound score of 0.70 ± 0.56 and 1.00 ± 0.74 in the high-power and low-power groups (*p* < 0.001), respectively, at 1 week, and a wound score of 1.96 ± 0.64 and 2.43 ± 0.59 in the high-power and low-power groups (*p* = 0.008), respectively, at 2 weeks, postoperatively. Regardless, complete wound healing was observed at the same time of week 4, on both sides, postoperatively (Fig. 3). Delayed postoperative bleeding occurred in 1 patient (3.8%) on the high-power side, but it was successfully controlled by applying direct pressure with gauze and performing a single cauterization using a bipolar cautery device under local anesthesia. There were no cases of major complications for re-operation or admission.

### Histological features of the surgical specimens

**Figure 4:**
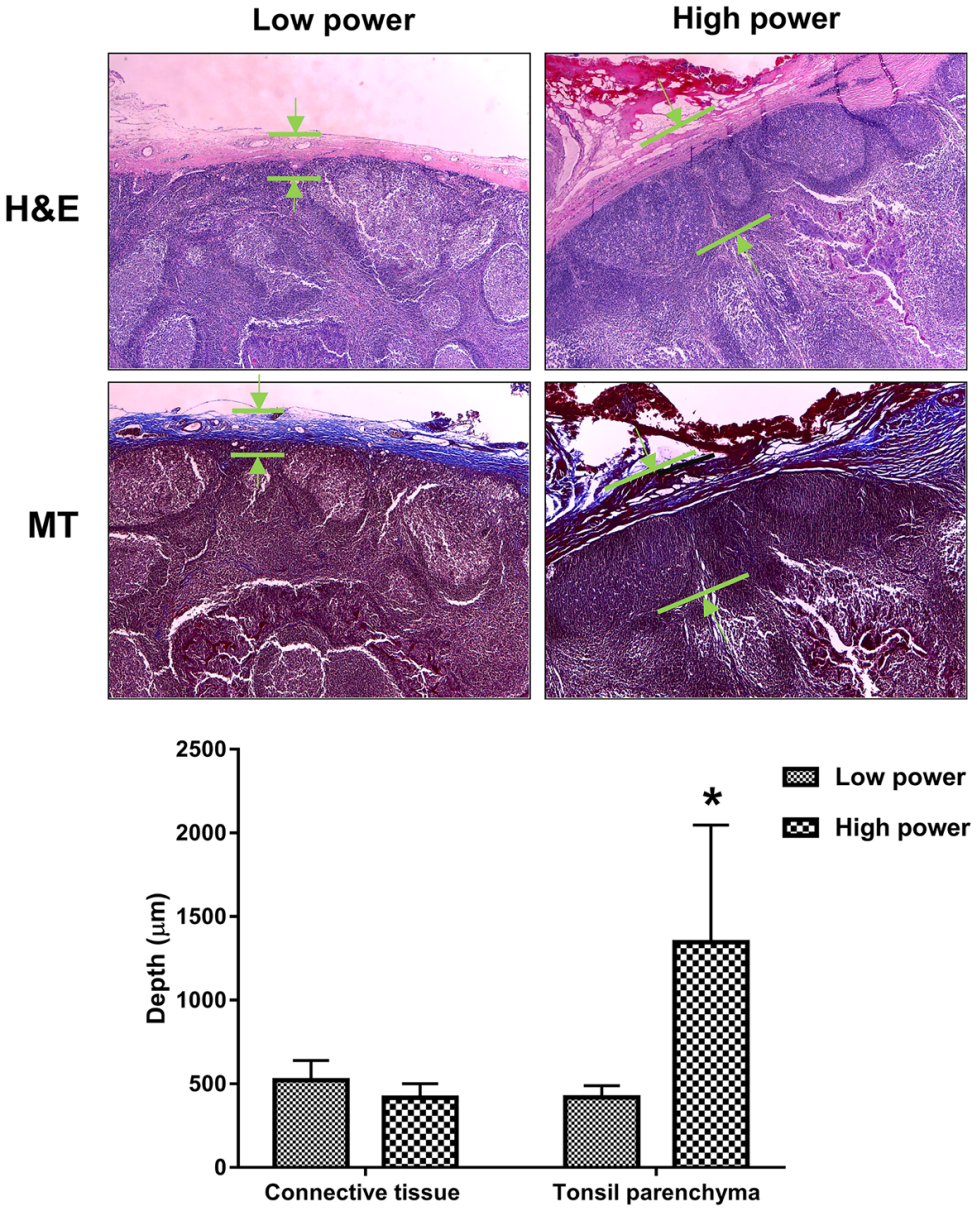
Hematoxylin and eosin and Masson trichrome staining for each resected tonsil. The degree of connective tissue shrinkage was greater on the high-power side than the low-power side. The depth of tissue damage observed on the resected surface of the tonsil parenchyma was significantly more extensive on the high-power side relative to the low-power side (green arrow). *P < 0.05. *H&E* hematoxylin and eosin, *MT* Masson trichrome.

Histologic features were compared among 20 specimens from 10 patients who agreed to provide a surgical specimen. The thickness from tonsil parenchyma of the resected connective tissue (between the tonsil and pharyngeal constrictor muscle) did not significantly differ, with a mean depth of 160.3 ± 39.5 µm and 136.8 ± 40.5 µm in the low and high-power groups (*p* = 0.83), respectively, but a greater degree of shrinkage due to electrical was observed on the high-power side than the low-power side. Especially, the damage to the tonsil parenchyma was significantly more extensive and deeper on the high-power side relative to the low-power side with a mean of 156.0 ± 36.8 µm and 565.2 ± 291.0 µm depth in both the low and high-power groups (*p* < 0.001), respectively. (Fig. 4).

## Discussion

There is a common belief among otolaryngologists that an increase in the diathermy energy used to carry out a tonsillectomy is associated with an increase in postoperative pain and wound healing^29^. This is thought to be due to a higher rate of tissue necrosis in the surgical plane, leading to an increase in postoperative pain and risk of delayed wound healing^1,30,31^. This study aimed to examine the effect of varying power settings in monopolar diathermy tonsillectomy on parameters such as operation time, postoperative pain, and wound healing. On the high-power side, the operation time was shorter, but the immediate and early postoperative pain was more severe, and wound healing was delayed. Pathologic findings showed that the damage to the tonsil parenchyma was deeper. Further, postoperative bleeding was more frequent on the high-power side. These findings suggest a dose–response relationship between the intensity of diathermy energy and the degree of damage to the surrounding surgical plane^12^.

The NICE guideline published in 2005 indicated that monopolar diathermy tonsillectomy, which had become popular with the development of ESU, may be somewhat dangerous in terms of postoperative bleeding compared to cold steel knife tonsillectomy. However, even today, tonsillectomy using monopolar diathermy is the most used in terms of the short operation time, which can reduce surgical complications, and operator convenience, and bipolar diathermy is often applied to control bleeding during surgery^4,11–18^.

Different surgical instruments are available for performing various types of tonsillectomy. These include cold steel knife and snare, monopolar and bipolar electro-surgery, carbon dioxide laser, and ultrasonic scalpel. Monopolar cautery is the most commonly used surgical method in tonsillectomy. Overall, this technique facilitates time-effectiveness, availability, and maneuverability. However, some limitations are associated with its use regarding thermal damage around the tissues, such as hemorrhage and wound healing. As new ESUs are developed, attempts to apply them to tonsillectomy continue to this day. When comparing tonsillectomy using diathermy to past cold knife surgery, the operation time is relatively short, but the incidence of secondary hemorrhage is reportedly high^32^. When the developed radiofrequency coblator was applied to tonsillectomy, the incidence of secondary hemorrhage was lower than that of diathermy^33^. Ultrasonic ESUs involve the application of a lower temperature than previous instruments, thus offering the advantage of minimizing tissue damage. Such devices are also associated with certain advantages regarding patient wellbeings, such as postoperative pain and wound healing periods^34–37^. Therefore, the electrical energy applied to the surgical plane is thought to influence both the patient’s operative and postoperative progress. However, in a study comparing the effect of different ESU powers on patients using the same instrument, rather than comparing different instruments, it was reported that the higher the power the shorter the surgery time. Therefore, if the ESU does not accurately measure the ‘on’ time on the surgical plane, there may be a difference in the actual applied electrical energy (power × time). In a study in which a timer developed by the author was applied to the foot switch of a bipolar diathermy device, the exact amount of energy applied to the surgical surface was determined by measuring the exact time that the actual bipolar diathermy was applied^38^. This clinical study also reported a higher tendency of postoperative bleeding at a high power.

Several studies have reported the effect of monopolar diathermy power on surgical wounds, but not tonsillectomy, according to the severity. When monopolar diathermy with different power settings was applied to the oral mucosa of mice, a narrower mucosal incision margin was observed at high power^39^. This may have the advantage of inducing more precise resection of the lesion by identifying a more precise boundary during surgery and shortening the operation time. Beak et al. reported that the high-power setting of electrocautery tonsillectomy was associated with a lower minimal hemorrhage rate^27^. However, since the operation time was significantly shorter in the group receiving high power, it is possible that perhaps the amount of energy (power setting × usage time) applied to the tonsil bed was erroneously small.

The basic mechanism of tissue resection using ESUs involves heating of the contact tissue via an electric current, followed by vaporization and ionization of the water content of tissue, and finally, vapor expansion and tissue fragmentation^40^. The amount of thermal energy and the current intervals are correlated with the degree of tissue damage^41,42^. The use of diathermy leads to heating of the tissue to an average temperature of 300–400 °C^43^. This mechanism is associated with postoperative pain, delayed wound healing, and scar contractures^44,45^. It is not clear why higher ESU power settings are associated with more severe postoperative pain and delayed wound healing. However, higher diathermy power settings may increase the area of tissue damage by thermal energy, which results in a larger area of tissue necrosis within the surgical plane. It was reported that the histological features of surgical specimens differed according to cold steel or diathermy dissection, and more damage was observed with diathermy dissection^21,46^. This can be expected to lead to postoperative pain and delayed wound healing.

However, this study has some limitations. First, this study included a small number of participants. To address this limitation, we exclusively enrolled adults who could accurately localize and express their own pain, as opposed to children. In addition, as an objective indicator, we assessed the wound healing scores and the extent of damage in the actual tonsil specimens. Second, it was not possible to measure the exact time when the ESU was applied, and the actual amount of applied energy was determined by multiplying the set power by the applied time. For accurate measurement, a timer-checking device should have been additionally applied to the ESU unit for measurement, but this was not possible because a suitable device could not be found. The time required for each operation on both sides was measured to determine the time that the instrument was applied, but this may have differed from the actual application time. Regardless, considering that the negative effect was significantly greater at high power despite a shorter operation time, the negative effect would have been more pronounced if the actual time was compared with the same. Additionally, the usual surgical method was resection of the tonsil with monopolar diathermy followed by the application of bipolar diathermy to the active and potential bleeding focus of the tonsil bed. Therefore, the effect of monopolar diathermy could be masked to some extent by the bipolar diathermy procedure for hemostasis on the tonsil bed. To prevent this potential masking, bipolar diathermy was applied with the same 15 W power and only to the same extent possible on both sides. However, these limitations must be considered when interpreting our results.

## Conclusion

We confirmed that using monopolar diathermy in a relatively low-power setting has more positive effects on the patient’s wellbeing, wound healing, and degree of tissue damage after tonsillectomy. Therefore, the surgeon should be aware that even if the operation time is slightly longer, it may be more beneficial to the patient to proceed with as low-power setting as possible during the operation.

### Supplementary Information

Supplementary Information.

## Data Availability

The datasets generated during and/or analysed during the current study are available from the corresponding author on reasonable request.
